# Exploring the mechanisms of host-specificity of a hyperparasitic bacterium (*Pasteuria* spp.) with potential to control tropical root-knot nematodes (*Meloidogyne* spp.): insights from *Caenorhabditis elegans*

**DOI:** 10.3389/fcimb.2023.1296293

**Published:** 2023-12-20

**Authors:** Keith G. Davies, Sharad Mohan, Victor Phani, Arohi Srivastava

**Affiliations:** ^1^ School of Life and Medical Sciences, University of Hertfordshire, Hatfield, United Kingdom; ^2^ Division of Nematology, Indian Agricultural Research Institute, New Delhi, India; ^3^ Department of Agricultural Entomology, College of Agriculture, Uttar Banga Krishi Viswavidyalaya, Dakshin Dinajpur, West Bengal, India; ^4^ Dr. D. Y Patil Biotechnology & Bioinformatics Institute, Dr. D. Y. Patil Vidyapeeth, Pune, India

**Keywords:** cuticle, surface coat, endospores, stem cells, seam cells, biological control

## Abstract

Plant-parasitic nematodes are important economic pests of a range of tropical crops. Strategies for managing these pests have relied on a range of approaches, including crop rotation, the utilization of genetic resistance, cultural techniques, and since the 1950’s the use of nematicides. Although nematicides have been hugely successful in controlling nematodes, their toxicity to humans, domestic animals, beneficial organisms, and the environment has raised concerns regarding their use. Alternatives are therefore being sought. The *Pasteuria* group of bacteria that form endospores has generated much interest among companies wanting to develop microbial biocontrol products. A major challenge in developing these bacteria as biocontrol agents is their host-specificity; one population of the bacterium can attach to and infect one population of plant-parasitic nematode but not another of the same species. Here we will review the mechanism by which infection is initiated with the adhesion of endospores to the nematode cuticle. To understand the genetics of the molecular processes between *Pasteuria* endospores and the nematode cuticle, the review focuses on the nature of the bacterial adhesins and how they interact with the nematode cuticle receptors by exploiting new insights gained from studies of bacterial infections of *Carnorhabditis elegans*. A new *Velcro-*like multiple adhesin model is proposed in which the cuticle surface coat, which has an important role in endospore adhesion, is a complex extracellular matrix containing glycans originating in seam cells. The genes associated with these seam cells appear to have a dual role by retaining some characteristics of stem cells.

## 1 Introduction

### 1.1 Plant-parasitic nematodes and biocontrol

Plant-parasitic nematodes are economically important pests that occur globally and constrain yields of both agricultural and horticultural crops (Jones et al., 2013; Dutta et al., 2019; Phani et al., 2021). Various management strategies that have been deployed to control these pests, including crop rotation, the use of various types of genetic resistance, flooding and the application of synthetic chemicals. Since the Second World War the use of synthetic chemicals has been a mainstay of phytonematode control, however, the recognition of pesticide toxicity to humans, domestic animals, non-target beneficial organisms and the environment has led to increasing legislation to prohibit their use in America, Europe and elsewhere. Therefore, the promotion for less hazardous approaches has been advocated (WHO, 2015). Biological control, involving the use of a pest’s natural enemies or hyperparasites, has long been recognised as a potential method to manage the plant-parasitic nematode pests; but the development of robust control strategies using such bioagents has always eluded crop protection scientists. It has been suggested that the lack of consistent control of nematodes lies in their biological variation, and the fact that their natural enemies and their nematode hosts are locked into a host-parasite arms race (Davies and Spiegel, 2011).

There are several groups of natural enemies that have the potential to be developed into microbiological control agents of phytonematodes (Stirling, 2014). These can be broadly characterised as: those that can be mass produced *in vitro* and can be grown on synthetic media, and those that are obligate parasites and can only be cultured *in vivo* within their hosts. Thus, they form two major groups either as the facultative microbes and the obligate microbes, respectively. Much research has focused on the use of fungi as many of them produce spores in synthetic culture media thereby extending their shelf-life and rendering them suitable for commercialisation. The bacteria, due to their diverse modes of nematicidal action, also show huge potential (Tian et al., 2007), but, with advancement in seed coating technologies (Rocha et al., 2019), which usually involve the incorporation of various fungicidal seed protectants, crop protection scientists have increasingly favoured the use of bacteria over fungi as nematicidal seed coatings.

### 1.2 *Pasteuria* as an alternative to nematicides

The *Pasteuria* group of bacteria are obligate Gram-positive parasites that infect invertebrates such as water fleas (*Daphnia* spp.) and nematodes including plant-parasitic nematode pests. Several species of *Pasteuria* have been characterized, all of which are obligate parasites, and they were originally placed within the family *Alicyclobacillaceae*. However, they have since been reclassified into their own family, the *Pasteuriaceae* (Vos et al., 2009). The taxonomic status of each group of *Pasteuria* species still remains obscure as it is currently mainly based on their hosts (Davies, 2009).

As the *Pasteuria* species all produce highly robust endospores that can remain dormant for many years (Giannakou et al., 1997), the bacterium thus proves to be ideal to be exploited as a biological control agent against a variety of phytonematode pests. And, indeed, early research had shown that *Pasteuria penetrans* (formally *Bacillus penetrans*) could effectively suppress and control the root-knot nematode *Meloidogyne javanica* (Stirling, 1984) through a combination of two mechanisms: (1) endospores adhere to the cuticle of the migratory second-stage infective juveniles (J2s), reducing their ability of the J2s to migrate and invade the plant root (Davies et al., 1991), and (2) when endospore encumbered J2s initiate a feeding site in a plant root, but before they moult into the J3s, the endospores germinate, form rhizoids that subsequently undergo exponential growth whilst simultaneously obliterating the nematode’s reproductive system (Davies et al., 2011; Phani and Rao, 2018).

However, following the publication of Stirling’s 1984 paper, it soon became apparent that endospores from one strain of the bacterium that attached to infective juveniles of one population of *M. javanica* would not attach to and infected other closely related populations of root-knot nematodes of the same species (Stirling, 1985; Davies et al., 1988; Espanol et al., 1997; Davies et al., 2001). Therefore, in practice there are two major challenges for the successful deployment of *Pasteuria* as a robust biological control agent and for commercial development. Firstly, as an obligate parasite, it will be necessary to mass produce it. Secondly it will be crucial to understand the nature of this host – parasite specificity initially focusing on the genetics and molecular biology underlying the biological variation of bacterial endospore – nematode cuticle compatibility. This article discusses the progress made to date in the development of *Pasteuria* as a biological control agent and suggest approaches for taking the research forward.

### 1.3 Host specificity in *Pasteuria*

The economically most devastating plant-parasitic nematodes are the sedentary endoparasites, i.e., the root-knot (*Meloidogyne* spp.) and the cyst (*Globodera* spp. and *Heterodera* spp.) nematodes (Jones et al., 2013). Both these nematode groups are parasitized by strains of *Pasteuria*, namely *P. penetrans* and *P. nishizawae* respectively. However, it is noteworthy that endospores of *P. penetrans* are unable to adhere to and infect the cyst nematodes and *vice versa*. Interestingly, the majority of endospore attachment bioassays have primarily focused on the attachment between *P. penetrans* and tropical *Meloidogyne* spp. that reproduce parthenogenetically and these studies (Stirling, 1985; Davies et al., 1988; Espanol et al., 1997; Davies et al., 2001) have revealed a high degree of host specificity. Conversely, the attachment bioassays between *P. nishizawae* and cyst nematodes, including *Globodera* and *Heterodera* spp., which reproduce amphimictically show a relatively broader range of host specificity (Davies et al., 1990; Sharma and Davies, 1996; Sharma and Davies, 1997) as compared to attachment studies involving *P. penetrans* and root-knot nematodes.

It has long been recognised that the evolution of sex in hosts can be accounted for by parasitic infections (Hamilton, 1980). This suggests that in amphimictically reproducing nematode species (e.g. *H. avenae*) there should be reduced *Pasteuria* infection. Conversely, in parthenogenetically reproducing species (e.g. *Meloidogyne arenaria, M. javanica* and *M. incognita*) there should be increased *Pasteuria* infection. Our examples above show that *Pasteuria* endospores that attached to and infected *H. avenae* germinated before the J2 had established its feeding site; these *Pasteuria* strains completed their sporulation within the J2 to produce in the order of 10^3^ mature endospores (Davies et al., 1990). Contrastingly, those *Pasteuria* strains that infect root-knot nematode species were delayed in their germination until the developing juvenile had established a feeding site and had more resources available for exponential growth and produced between 1 x 10^6^ and 2 x 10^6^ endospores per individual nematode (Davies et al., 1988), more than a thousand-fold increase in mature endospores. It appears that there is an interesting trade-off between the parthenogenetically reproducing nematodes, i.e. the tropical root-knot nematodes (*Meloidogyne* spp.) with *P. penetrans*, which exhibited delayed germination and its higher endospore production, versus the temperate *H. avanae* amphimictic cyst nematode infected with a *Pasteuria* strain that germinate early in the J2 resulting in far fewer mature endospores.

Assuming that delayed germination evolved later than immediate germination, this appears to contradict the view that amphimixis leads to reduced parasitism and parthenogenesis results in increased parasitism. Conversely, if immediate germination evolved earlier than delayed germination, this would be concordant with the idea that amphimixis leads to reduced parasitism. The paper by Hamilton (1980) discusses several scenarios in which reproductive modes may diverge from the view that amphimixis reduces parasitism. It suggests that the intensity of selection may vary due to the number of loci involved and other frequency-dependent factors. Interestingly, analysis of 19 genomes, representing five key species of tropical parthenogenetic *Meloidogyne* populations, revealed them to be the result of the hybridization of two divergent genomes, which generated species divergence through dynamic non-crossover recombination, generated species divergence (Szitenberg et al., 2017). Following Hamilton (1980)’s argument this might suggest that the effects of *Pasteuria* parasitism on the evolution of parthenogenetic species (*Meloidogyne* spp.) may be the result of fluctuating linkage disequilibria that is less likely in amphimictic (*Heterodera* and *Globodera* spp.) species.

The coevolution between *Caenorhabditis elegans* and the pathogen *Serratia marcescens* has been found to demonstrate, following the Red Queen hypothesis, that selection favoured outcrossing mixed mating populations rather than obligate selfing populations (Morran et al., 2011). The first interaction between endospores of the bacterium and the cuticle of the nematode is the process of endospore adhesion and it would therefore be of interest to apply this hypothesis to the *Pasteuria* endospore – nematode cuticle model. The molecular mechanism of endospore attachment onto the nematode cuticle and the genes involved is therefore a key first step in understanding this process. This review will explore the co-evolutionary arms races between endospore adhesins and the cuticular receptor using insights gained from *C. elegans.*

## 2 The mechanism of endospore attachment

### 2.1 Biochemistry of adhesins

Early research on the nature of *Pasteuria*’s adhesins focused on the biochemical characterization of their endospores (Persidis et al., 1991). This work recognised that disrupted exosporia and spore fragments of *P. penetrans* were fibrous and although no longer intact these fragments were able to retain their host-specific attachment to the nematode cuticle of infective juveniles. Subsequent bioassays involving endospores pretreated with selected proteases and glycolytic enzymes reduced endospore attachment suggesting that the adhesins were composed of a combination of proteins and carbohydrates (Davies and Danks, 1993). In addition, endospores pretreated with polyclonal antibodies raised to whole spores also reduced attachment (Persidis et al., 1991). Interestingly, monoclonal antibodies revealed a heterogeneity on the surface of the endopsores that was specific to the population of root-knot nematode to which they attached (Davies et al., 1994; Davies and Redden, 1997). If *Pasteuria* is to be developed into a biological control agent, it will be necessary to understand the nature of this specificity so that endospores can be successfully deployed to control plant-parasitic nematodes. Comparative phylogenetic analysis of *P. penetrans* based on *spo0A*, a gene that plays a key role in the initiation of endospore formation, suggested it was closely related to the *Bacillus* group (Trotter and Bishop, 2003). In a subsequent phylogenetic analysis, following a genomic sequencing survey and using multiple genetic loci, *Pasteuria* appeared to be a member of the *Bacillus – Clostridium* clade (Charles et al., 2005).

### 2.2 The collagen-like fibrous nap of endospores

In the early noughties, the genomes of bacteria responsible for diseases in both humans and animals, as well as those of other close relatives, were a primary goal of sequencing projects. Within this group of genomes, of which the endospore forming *Bacillus* spp. were important, the new sequence analyses were also being used to investigate the development, construction and viability of endospores. A comparative analysis of the rhamnose cluster operon between *B. cereus* and *B. anthracis*, revealed eight genes that were identified as likely to encode for the outer structural components of the endospore such as bacterial collagens (Todd et al., 2003). Bacterial collagen-like proteins have become an important area of study (Qiu et al., 2021) and several of these genes present in the animal pathogenic bacteria *B. cereus* and *B. anthracis*, are absent in *B. subtilis*, including the glycoprotein BclA that contains a GXX motif. The glycoprotein BclA encodes for collagen-like fibres which forms a hair-like nap on the surface of the endospore; the length of this nap is related to the number of repeats of the GXX motif (Sylvestre et al., 2002; 2003). Several homologs to these endospore genes from the rhamnose cluster operon were identified in genome survey sequences of *Pasteuria penetrans* using BLAST including sequences similar to BclA (Schaff et al., 2011; Orr et al., 2018). A hair-like nap is also associated with the outer surface of the endospores of *P. penetrans*, and it has been proposed that these fibres are possibly responsible for their attachment of endospores to the infective juvenile of the nematode through a *Velcro*-like attachment process (Davies, 2009). A total of 17 different putative collagen-like encoding regions, revealing a high degree of potential diversity, were identified from *P. penetrans*, strain 148, which had a restricted host range but revealing a high degree of potential diversity (Srivastava et al., 2019). Similar diversity has been observed in the closely related *Pasteuria ramosa*, which is a parasite of *Daphnia* spp. (Mouton et al., 2009; McElroy et al., 2011).

The observation that these collagen-like genes are highly diverse, as revealed by the monoclonal antibodies and their link with host attachment (Davies et al., 1994), provides further evidence that they could possibly be a determinant of host range. However, the majority of studies looking at the mechanism of endospore attachment have primarily relied on loss of function experiments that involved treating endospores with heat, enzymes, lectins and antibodies (e.g. Davies et al., 1988; Davies and Danks, 1993; Davies and Redden, 1997) and then measuring changes in the number of endospores adhering to host juveniles in attachment bioassays. As *Pasteuria* are a group of bacteria that are obligate parasites and cannot be grown *in vitro*, the ability to produce knockouts is problematic. Therefore, rescue experiments, in which gain-of-function studies would be an ideal way of identifying function, become impossible. An alternative approach would be to identify a closely related bacterium that can be grown *in vitro* and has the genetic apparatus to produce endospores. Clearly, *Bacillus subtilis* could fulfil these criteria, but as discussed above, it does not have a sufficiently intact rhamnose cluster operon as contained within the genomes of animal parasitic bacilli *B. cereus* and *B. anthracis* (Todd et al., 2003). Therefore, another approach would be to select an animal parasitic bacterium, for example *Bacillus thuringiensis*, which produces endospores with a hair-like fibrous nap but does not represent a threat to humans or domestic animals (Srivastava et al., 2022).

### 2.3 Similarities between *Pasteuria* and *Bacillus thuringiensis*

Recent comparisons between endospore protein extracts of several strains of *B. thuringiensis* and *P. penetrans* revealed 25 proteins with various molecular weights. Among these, only one band at 58 kDa was common to all *B. thuringiensis* and *P. penetrans* (Srivastava et al., 2022). Six proteins, at 150, 34, 30, 24, 17 and one > 9 kDa were common across all *B. thuringiensis* strains with the remaining 18 proteins having variable distributions across all bacterial strains (Srivastava et al., 2022). Interestingly, the same study using two antibodies raised to two short collagen-like synthetic peptides from *P. penetrans* that recognised the outer endospore coat revealed two glycoproteins (> 250 kDa and ~72 kDa) that when treated with collagenase were digested. Attachment bioassays using *Pasteuria* endospores treated with the same collagenase also reduced attachment to second-stage juveniles. This suggests that the outer endospore coat of *B. thuringiensis* spores, although they do not adhere to the cuticle of infective juveniles of root-knot nematode, they do share biochemical properties with *P. penetrans* and could perhaps be used as a model for gain of function endospore attachment assays (Srivastava et al., 2022).

## 3 Nature of the cuticle receptor

### 3.1 The *Caenorhabditis elegans* model

The cuticle forms the nematode’s exoskeleton and performs a number of functions: it is important in determining the nematode’s morphological integrity, and it forms the major barrier between the internal body structure of nematode and its external environment; it acts as a gatekeeper regarding molecular permeability; it is important for vermiform locomotion; and it forms a barrier against microbial pathogens. At each stage of the nematode’s development when it undergoes ecdysis the cuticle gets remade anew, but there are very few comparative studies that examine the structure of the different stages. The most studied nematode cuticle is that of the adult stage of *Caenorhabditis elegans* (Page and Johnstone, 2007), which increasingly became a model for investigating the host-microbial interactions (Gravato-Nobre and Hodgkin, 2011). The adult cuticle has a complex structure which is secreted by the hypodermis and forms a part of the extra-cellular matrix; this matrix consists primarily of collagens together with insoluble cuticlins, glycoproteins and lipids.

Early studies of the cuticle using ethyl methanesulfonate (EMS) as a mutagen affected the ability of selected lectins to recognise the cuticle surface. These mutants were called the *Srf* mutants (Politz et al., 1990; Link et al., 1992), and the genes involved are increasingly being recognised as important in microbial pathogenesis. The mutant phenotypes with altered lectin binding characteristics became associated with several different *C. elegans* pathogens (Gravato-Nobre et al., 2005; Darby et al., 2007; Gravato-Nobre et al., 2011). Subsequent more recent studies have expanded and characterized a range of other *C. elegans* mutants which alter various phenotypic traits (e.g. *Bah*, biofilm absent from head; *Bus*, bacterially unswollen; *Dar*, deformed anal region; *Gro*, slow growth; *Hbp*, head biofilm present; *Skd*, skiddy locomotion). The majority of these genes that are responsible for altered pathogenicity in several different bacteria such as *Microbacterium nematophilum*, *Yersinia pseudotuberculosis* and strains of *Leucobacter* spp., and are associated with an altered cuticular surface coat (SC) (O'Rourke et al., 2023). The cuticle SC, or glycocalyx, is rich in lipids and glycoproteins, the source of which is increasingly thought to be the seam cells (Gravato-Nobre and Hodgkin, 2011; O'Rourke et al., 2023). These seam cells lie beneath the lateral mid-line and have an important role in regard to producing a diverse range of complex compounds that affect the ability of microorganisms to adhere and form biofilms.

### 3.2 Mucins and nematode parasites

The SC of the animal parasitic nematode *Toxocara canis*, which primarily affects domestic dogs and other canids but can also affect humans, has been shown to contain a mucin which is important in the evasion of the host’s immune response (Page et al., 1992). It has long been recognised that mucins play an important role in host-parasite interactions, including those involving helminths (Hicks et al., 2000; Theodoropoulos et al., 2001). Mucins appeared very early in metazoan evolution and are proteins high in proline, serine and threonine, with a high molecular weight in which their carbohydrate content is O-linked to the amino acids serine and threonine (Lang et al., 2007). Up to 50 percent of the molecular weight of a mucin can be carbohydrate, which structurally makes them a hugely diverse group and therefore they are an ideal molecule to protect nematodes from initial microbial attachment and biofilm formation as they migrate through the soil (Davies and Curtis, 2011). Lang et al. (2007) identified a number of mucin-like proteins with homologous sequences present in *C. elegans*; these sequences were also present the phytonematode species *M. hapla* and *M. incognita* (**Table 1**).

**Table 1:** *Caenorhabditis elegans* mucin-like proteins and their respective RNAi clones with BLASTP hits to *Meloidogyne hapla* and *Meloidogyne incognita* together with their percentage positive identity.

Mucin-like Gene (name)^*^	RNAi Clone^#^	Blastp hits E-value	
		*M. hapla* (% Id^$^)	*M. incognita* (% Id^$^)
F59A6.3 (mucl-1)	II 4M05	2.0e-61 (58)	7.0e-33 (42)
C12D12.1 (mucl-2)	X 2C10	6.0e-43 (53)	5.0e-32 (48)
F16F9.2 (dpy-6)	X 4G10	6.0e-60 (52)	3.0e-35 (43)
H43E16.1 (mucl-6)	II 5K17	1.0e-66 (41)	5.0e-46 (44)
	II 5I21		
	II 5M01		
K06A9.1 (mucl-9)	X 1B17	1.0e-60 (45)	2.0e-72 (41)
H02F09.3 (mucl-10)	X 1B23	1.0e-80 (43)	3.0e-52 (38)
C26G2.2 (mucl-11)	X 6F18	5.0e-17 (44)	2.0e-12 (43)
C07G2.1 (cpg-1)	III 2C06	2.0e-14 (57)	1.0e-05 (46)
K11D12.1 (cwp-4)	V 3J24	2.0e-22 (54)	2.0e-20 (53)
	V 3L02		
	V 4C07		
F35E12.7 (dct-17)	V 9E05	1.0e-21 (48)	4.0e-17 (48)
C29E6.1 (let-653)	IV 6 G10	1.0e-55 (80)	1e-101 (78)

^*^Mucin-like proteins identified from Lang et al., 2007; and the linked website:

www.medkem.gu.se/mucinbiology/databases/.

^#^From the C. elegans RNAi library of Kamath and Ahringer (2003).

^$^Blastp percentage positive identity.

Recent work with the plant-parasitic root-knot nematode (*Meloidogyne* spp.) has used RNAi knockdown methodology to investigate a range of potential cuticle and SC-related proteins to understand their role in *P. penetrans* attachment (**Table 2**). A functional study of a mucin-like protein characterized from *M. incognita*, designated *Mi-muc-1*, was found to be rich in serine and threonine and highly expressed in the pre and post parasitic second-stage juvenile (J2) and the pre-egg laying female (Phani et al., 2018b). *In situ* hybridization studies revealed expression in the tail region of the J2 around the phasmid. Subsequent knockdown experiments using dsRNA designed for *Mi-muc-1* significantly reduced endospore attachment. Further experiments designed to identify the carbohydrate domains involved were shown to be D-glucose, D-galactose and D-xylose, whereas other sugars also tested (D-fructose, D-mannose, L-arabinose, L-sorbose) had no or little effect. Although perhaps this result might not be unexpected if mucins are a component of the nematode cuticle surface, it is interesting that other unrelated genes have also been shown to affect the attachment of *P. penetrans* endospores to J2 cuticle.

**Table 2:** Effects of RNAi knockdown experiments of genes known to be important in nematode cuticle and/or cuticle surface coat of *Meloidogyne incognita* due to their effects on *Pasteuria penetrans* endospore attachment to infective juvenile cuticle.

Gene Knockdown	Protein role	*Pasteuria* endo-spore attachment	Reference
Mucin-1 protein (*Mi-muc-1*)	Host-parasite interaction	decrease	Phani et al., 2018a
Selenium-binding protein (*Mi-SeBP-1*)	Environmental, biotic and abiotic stress related	increase	Phani et al., 2018b
Fatty-acid and Retinol-binding protein (*Mi-far-1*)	Nutrient acquisition development and reproduction	increase	Phani et al., 2017

### 3.3 Other cuticle proteins

Fatty acid retinol binding proteins are unique to nematodes. They play an important role in nutrient acquisition and immune response being present in the nematode cuticle (Kennedy et al., 1997; McDermott et al., 1999; Prior et al., 2001; Garofalo et al., 2003; Iberkleid et al., 2013). Originally identified in animal parasites, they have been shown to be increasingly important in plant parasites and have been shown to affect the endospore binding of *P. penetrans* onto J2 cuticle (Phani et al., 2017). A fatty acid retinol binding protein that was cloned from *M. incognita*, designated Mi-FAR-1, was found to be rich in α-helix structure and contained both a casein kinase phosphorylation and a glycosylation site was characterized. Its expression was observed in all developmental stages, with the highest expression appearing in the fourth stage juvenile. *In situ* hybridization studies of *Mi-far-1* revealed its expression in the hypodermis of the J2 cuticle that when silenced showed an increase in endospore attachment. This suggests that Mi-FAR-1 may have a protectant role in inhibiting the *Pasteuria* endospore attachment.

Another protein of the fatty acid binding protein (FABP) superfamily, the selenium binding protein Mi-SeBP-1 was also expressed in the nematode hypodermis and also increased *Pasteuria* endospore attachment on being silenced (Phani et al., 2018b). The protein was identified as important in a differential expression study between *Pasteuria* encumbered and unencumbered J2s of *M. incognita* and was the first characterization of a selenium binding protein in nematodes. Although knockdown experiments were found to affect endospore attachment, it had no observable effect on the nematodes ability to invade roots, nor did they subsequently affect nematode fecundity. This particular protein shares a 34% identity to a *C. elegans* selenium binding protein which was orthologous to a human selenium-binding protein (SELENBP1), a regulator of lifespan and stress resistance (Köhnlein et al., 2020). It is therefore perhaps not surprising that knockdown experiments in root-knot nematodes led to increased endospore attachment.

### 3.4 Role of seam cells

There is increasing evidence that the origin of the cuticle surface coat is associated with the hypodermis and its associated seam cells. Noteworthy is the fact, that silencing experiments with both the *Mi-far-1* and *Mi-SeBP-1* led to an increase in endospore attachment, whereas knockdown of *Mi-muc-1* decreased the endospore binding. From these findings, it can be presumed in a generalized layout that the members of FABP superfamily proteins being associated with the nematode hypodermis might act as protectants against microbial pathogenesis, whereas the glycosylated mucins or mucin-like proteins act as facilitators. The FABPs are reportedly involved in innate immunity and antimicrobial responses in other invertebrates (Cheng et al., 2013; Tan et al., 2015; Wang et al., 2017). But, the mucin-like protein being a basic structural component for cuticular integrity when facilitates the pathogenesis it indicates a co-evolutionary advancement for the obligate bacterium *Pasteuria* that targets a basic cuticular constituent for its secured parasitic success at the stage of attachment. The hypothesis can be further strengthened with the red blood cell (RBC) attachment assay results (Phani et al., 2018a), where the soaking of *M. incognita* J2s into different carbohydrates (stated above) showed negligible effect on RBC attachment, but significantly affected the endospore attachment. If we assume that both the endospores and RBCs use glycan-mediated support for their attachment, getting differential attachment with same carbohydrate molecule is an indicative of involvement of different ligands for the endospores and RBCs, which may have possibly developed during the co-evolutionary arms race of *Meloidogyne* with *Pasteuria*, but not the RBC.

Reviewing the work of (Phani et al., 2017; Phani et al., 2018a; Phani et al., 2018b) with *M. incognita*, there was clear evidence that *Mi-FAR-1*, *Mi-SeBP-1* and *Mi-muc-1* were all associated with hypodermal expression, but nothing to specifically to link them to seam cells. However, and interestingly, most of the genes identified in *C. elegans* involved in bacterial resistance were expressed by seam cells (O'Rourke et al., 2023) which are known to be hypodermal, located along each side of the nematode beneath the alae, and undergo repetitive replacement during the nematode’s development (Page and Johnstone, 2007). Only *Mi-muc-1* and *Mi-SeBP- 1* were primarily investigated by Phani et al. (2018a; 2018b) for their association with bacterial infection, the other, *Mi-FAR-1* was primarily associated with retinol acquisition and had been shown to affect the interaction between the nematode and its plant host (Iberkleid et al., 2013). The fact that *Mi-muc-1* is highly glycosylated and was the only gene linked to a decrease endospore attachment, whereas both the others were comparatively poorly glycosylated and both increased endospore attachment, may suggest *Mi-muc-1* to be directly related to microbial resistance and possibly seam cell related (although not reported in Phani et al., 2017), while the others, *Mi-FAR-1* and *Mi-SeBP- 1*, the increase in endospore attachment is an indirect consequence of knockdown.

## 4 Multitrophic interactions

### 4.1 Root exudates

It has long been recognised that exposure of certain groups of plant-parasitic nematode to plant root exudates, in particular potato cyst nematodes *Globodera rostochiensis* and *G. pallida*, have a variable effect on egg hatch (Evans, 1983). More recently, exposure of J2s of *M. arenaria* to root exudates from eggplant, *Solanum melongena*, led to a decrease in endospore attachment compared to controls, irrespective of whether plant host or plant non-host exudates were used (Liu et al., 2017). Conversely, although not unequivocally, in a study of exposure of J2s from *M. incognita* and *Heterodera cajani*, each with its own homologous specific *Pasteuria* population, it was shown that as the J2s aged, they developed an increasing resistance to their respective homologous endospore populations (Mohan et al., 2020). However, if the J2’s were exposed to root exudates of their host plant prior to a spore attachment bioassay, the rate of increasing resistance to endospore attachment as the J2s aged was reduced. In contrast, when exposed to root exudates of a non-host plant, this effect was not observed. Subsequent analysis of the plant root exudates by GC/MS could not identify any obvious compounds that could be associated with the observed results. However, evidently, the root exudates were affecting the aging process of the cuticle which was interpreted as a tritrophic interaction to help recruit the bacterium to the plant’s long-term benefit (Mohan et al., 2020).

### 4.2 Cuticle surface coat

The variable effects observed in endospore attachment from RNAi knockdown experiments of FAR, SeBP and the mucin reported by (Phani et al., 2017; Phani et al., 2018a; Phani et al., 2018b), together with the effects of the plant root exudate experiments (Mohan et al., 2020) suggests that endospore attachment to J2 cuticle receptor is complex. The results are not only the result of the co-evolutionary arms race between the bacterial adhesins and the nematode cuticle receptor/s over inter-generational time, but also of specific spatial trade-offs between differentially expressed surface coat compounds along the length of the nematode itself. For example, Spiegel et al. (1996) reported the treatment of *M. javanica* J2s with certain lectins differentially affected endospore attachment along the length of the juvenile where attachment to the overall body could be distinguished from the head region. The current author (KGD) has witnessed similar spatial differences in endospore attachment in *M. hapla* (personal communication). This clearly indicates that the proportional expression of a particular gene, or genes, in one area is different from a particular cuticular compound in another area. Therefore, the balance of the expression of these endospore-attractive and endospore-repulsive molecules will determine the overall number of endospores adhering to the cuticle in any particular region, and this is mediated by the cuticle SC. The SC is evidently an arrangement of many complex macro-molecular compounds and the additional observation that root exudates clearly influence spore attachment through altered maturation begs the question as to the signalling processes involved and the role that seam cells may or may not play in the cuticle aging and endospore attachment process.

Joshi et al. (2010) report that there are parallel modes of production and proliferation of cell lineages in stem and seam cells of *C*. *elegans*. In *C. elegans*, true stem cells are associated with the distal tip cells of the ovary where they are maintained in a proliferative state prior to differentiation during embryogenesis. This is important as they retain the ability to maintain pluripotent plasticity with a potential for future cellular commitment. These same self-renewal and expansion patterns in stem cells, although poorly understood, are mimicked by the lateral epidermal seam cells (Joshi et al., 2010). It is therefore possible that the genes in stem cells that are responsible for the production of complex surface cellular compounds and important in molecular differentiation and cellular commitment are the same as those genes expressed in seam cells and are the origin of the surface coat of the cuticle that affects endospore adhesion.

Here, seam cells become key because they are an origin of the SC and important in the expression of mucins which are complex molecules that have a role in bacterial infection processes (Parsons et al., 2014; O'Rourke et al., 2023). Noteworthy, is that in *C. elegans* the *bus*-8 gene, which encodes a glycosyltransferase, appears to have a dual role in epidermal morphogenesis. Firstly, it is involved in the migration of epidermal cells during embryonic ventral closure, and secondly, it plays an important role in the adult by producing a host surface receptor that makes the nematode susceptible to the bacterium *Microbacterium nematophilum* and to which the bacterium can bind (Partridge et al., 2008). It is interesting to speculate on the number of dual roles these genes associated with building complex molecules on the cuticle SC may have. For example, has the functional plasticity which is maintained in stem cells and is important in the cellular organisation and differentiation of the developing embryo, through expression in the extracellular matrix (ECM), been co-opted for generating cuticle SC diversity? And can it, therefore, also act as a receptor to which bacteria can attach to the cuticle and, by its regulation, either generate SC diversity or altered cuticle maturation?

### 4.3 The extracellular cuticle matrix

The cuticle of *Caenorhabditis elegans* has been postulated to be a model for ECM (Page and Johnstone, 2007) where, as stated above, it plays a fundamental role in providing a flexible and resilient barrier to the nematode’s environment and a site for microbial infection. Stem cells also are surrounded by an ECM, which provides a physical support environment and is a non-cellular component of the cells, mainly made up of proteins and polysaccharides (Frantz et al., 2010). In *C. elegans* it regulates overall cellular differentiation from the single cell to the adult, including now more fully understood specific roles such as pharyngeal development, embryo elongation, and vulval formation (Kelley et al., 2015; Walma and Yamada, 2020). As well as containing a number of fibrillar molecules like collagen and elastin, ECM also contains a large number of other components with which they interact including surface receptors, e.g. integrins and fibronectin, and growth factors, e.g. TGFβ and interleukins (see **Table 1**; Walma and Yamada, 2020). However, it is becoming increasingly clear that a diverse number of mechanisms are responsible for stem cell fate, in which environmentally determined niche signals go beyond carbohydrates and biochemical signal transduction pathways. These mechanisms can also include subtle changes in physical forces, for example those that may involve changes ECM stiffness (Watt and Huck, 2013).

It has been known for some time that specific glycans are limited to different developmental stages of *C. elegans*; for example, phosphorylcholine can be highly decorated with stage specific complex oligosaccharides (Cipollo et al., 2005), and the glycosaminoglycan chondroitin sulphate, which comprises of a pair of repetitively linked N-acetylgalactosamine and glucuronic acid molecules, is commonly found to glycosylate molecules found in the ECM (Izumikawa et al., 2004). Experiments blocking chondroitin synthesis in *C. elegans* resulted in defects in early embryogenesis, and eventually prohibiting cell division; however, rescue experiments with PAR2.4, the worm homolog of human chondroitin synthetase which acts as a neural stem cell in young embryonic lineages, was also found to be expressed in seam cells (Izumikawa et al., 2004). It, therefore, seems reasonable to hypothesise that PAR2.4, like *bus-8* (Partridge et al., 2008), may also have a dual role; firstly, in the fate of neural stem cells during embryogenesis, and secondly, to have a function in nematode cuticle SC, possibly even having different roles at the various life stages of the nematode during ecdysis, of which we currently know very little. Interestingly, beneath the alae, which form longitudinal ridges down each side of the nematode and are restricted to the L1, dauer, and adult life stages of *C. elegans*, are the seam cells, which are biochemically and ultrastructurally distinct (Page and Johnstone, 2007).

Do seam cells, therefore, have a role in which they have retained some functional properties of stem cells which have been co-opted for use in later life-stages of the nematode life-cycle? And if so, for what use? The most abundant of our knowledge of the nematode cuticle is of the adult cuticle, which is spatially the largest and most easily obtained for study in the case of *C. elegans*. Arguably, the longest-lived developmental stage of *C. elegans* is the dauer-stage larvae, but little research other than descriptive ultrastructural studies have been done. However, in plant-parasitic nematodes the second-stage juvenile (J2) is often regarded as a dauer-like stage; it morphologically has hypodermal seam cells covered by alae, and it is non-feeding as it needs to locate and infect a host plant first. Here, we want to put forward the hypothesis that the biochemical diversity of the ECM of stem cells has been retained by seam cells to generated biochemical diversity of the nematode cuticle ECM; this diversity may play a role in the tritrophic interactions between obligate plant-parasitic nematodes and their cuticle pathogens/parasites. This has recently been exemplified by the studies between plant-parasitic nematodes and *Pasteuria*, which demonstrates not only temporal co-evolutionary changes in the field related to crop rotation (Liu et al., 2018), but also developmental changes related to the rate of maturation of the cuticle SC of the J2 (Mohan et al., 2020). This demonstrates the possibility of genetic pleiotropic effects that cross-connect multiple levels of biological organisation from germline stem cells to adult cuticle, the latter of which can be affected by plant root exudates.

## 5 Implications for phytonematode control strategies

Since the publication of *Silent Spring* by Rachel Carson in 1962 there has been increasing concern over the use of broad-spectrum chemical pesticides (van Emden and Peakall, 1996), a concern that continues to this today. Nematicides are no exception (Davies and Spiegel, 2011) and climate change, along with the increasing global threats to ecosystem health, has only exacerbated these concerns (Fisher et al., 2012; Dutta and Phani, 2023). Biological control and the use of a pests’ natural enemies has remained an active area of research as one among several potential solutions. Here, we have demonstrated that the host-parasite interactions are complex and can be viewed from several differing perspectives, that range from specific host – parasite genetic interactions that produce co-evolutionary arms races between two organisms, to multiple cross-connected trophic interactions that affect the population dynamics of the ecological communities involved. For biological control to be used as a robust management strategy for phytonematodes, the outcomes of these multitrophic interactions are required to result in an ecologically resilient community suppressive to the pest nematodes. Although this approach was successful in controlling the cereal cyst nematode *H. avanae* through a field-based monocropping approach (Gair et al., 1969) that exploited soil microbial diversity as an ecosystem service (Gair et al., 1969; Stirling, 2014), this has never been successfully and robustly implemented in the field through the deployment of microbes. While there are numerous examples of the addition of single organisms to control plant-parasitic nematodes in highly managed glasshouse experiments, it has proved notoriously difficult and indeed elusive in the field, and requires broad-based knowledge at the population, organismal and molecular scales (Kerry, 2000).

**Figure 1:**
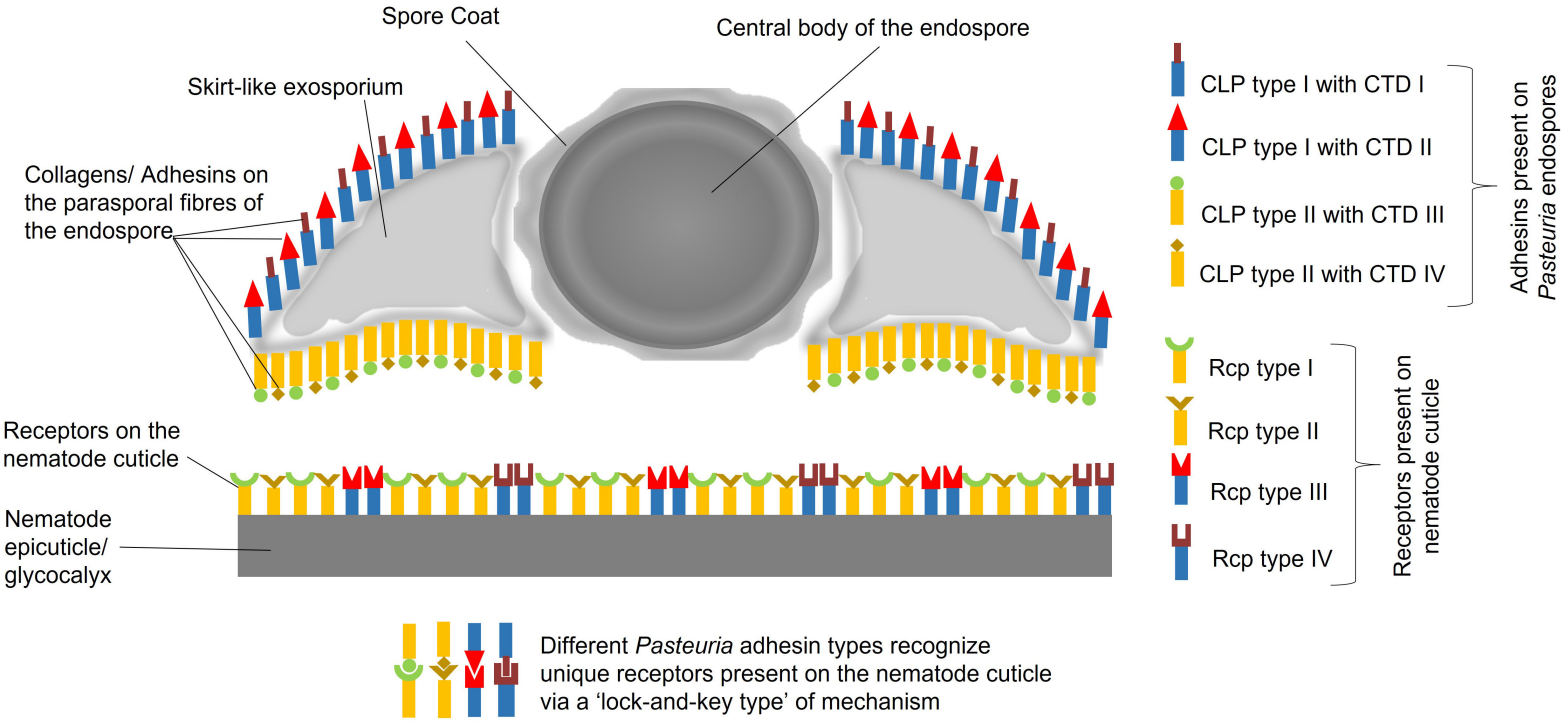
Multitype Adhesin Model, postulating an array of adhesins that adorns the surface of *Pasteuria* endospores, comprising diverse array of collagen-like proteins, denoted as CLP I, CLP II, CLP III, CLP IV, and so forth. These CLPs, exhibit varying distributions on the concave and convex sides of the endospores. Each CLP possesses a unique C-terminal domain, referred to as CTD I, CTD II, CTD III, CTD IV, etc., which specifically recognizes distinct receptor types on the nematode cuticle. The cuticle receptors (Rcp type I, II, III and IV) being the product of either mucin-like genes possibly expressed in the seam cells and fatty acid retinol binding proteins (FABPs) superfamily, a product of genes expressed in the hypodermis. Consequently, there are various possible combinations of CLPs, CTDs, and their corresponding cuticle receptors (Rcp type I, II, III and IV). Some CLPs may even share common CTDs by their sharing common glycoconjugates (like NAG) that exhibit variations among different CLPs located on either side of the endospore.

Here, in order to build coherence between the functional, ecological and molecular levels of interactions, we have dissected the intricate relationships between the obligate Gram-positive bacterium *Pasteuria* spp. and the tropical group of root-knot nematodes *Meloidogyne* spp. gaining insights from *C. elegans.* Previously, it was hypothesised that a *Velcro*-like attachment process was one of the key factors underlying the mechanism by which endospores of the bacterium attached to the cuticle of the nematode (Davies, 2009). Over the last 10 years subsequent research, as outlined above, has shed light on some of the details of this *Velcro-*like interaction (**Figure 1**). In this multiple adhesin model (Srivastava, 2017), various molecules on *Pasteuria* endospores interact, through a ‘Velcro-like mechanism’, with different receptor molecules on the nematode cuticle. Electron micrographs show these adhesins to be unevenly distributed with a greater density on the concave surface rather than the convex surface of the endospore, (Davies, 2009) showing there are more mechanisms of generating adhesin diversity beyond collagen length, carboxyl-terminal sequence variation and glycosylation. The nature of the cuticle receptor is likely to be equally capable of also generating a level of molecular diversity. Here, we suggest that the cuticle receptor is a trade-off between two components. Firstly, the biochemical nature of the cuticle as part of the extracellular matrix as secreted by the hypodermis, and secondly, the biochemistry of the cuticle’s surface coat produced as a secretion from the seam cells. We propose that this diversity may be partially maintained by the ability of seam cell gene expression which has retained a dual role by maintaining stem cell plasticity and diversity that played an important role in embryogenesis.

The *Pasteuria*-nematode interaction is complex, requiring a broader perspective to fully comprehend the molecular mechanisms involved. In the context of the Red Queen Hypothesis, which describes an ongoing evolutionary arms race between hosts and their pathogens. Our model suggests a dynamic co-evolutionary relationship in which *Pasteuria* has developed a range of adhesins, including collagen-like proteins, to attach to a range of different receptors on the nematode cuticle. This can be seen as an adaptation by *Pasteuria* to ensure successful attachment and infection in the face of co-evolving nematode defence mechanisms. The genes coding for these diverse glycosylated adhesins on the surface of the endospore and the biochemical nature of this co-evolving receptor specificity is currently far from being understood and remains a matter of speculation and the basis for imaginative experimentation if novel control strategies are to be developed.

## Author contributions

KD: Conceptualization, Project administration, Supervision, Writing – original draft, Writing – review & editing. SM: Conceptualization, Methodology, Supervision, Writing – review & editing. VP: Conceptualization, Investigation, Methodology, Writing – review & editing. AS: Conceptualization, Investigation, Methodology, Writing – review & editing.
